# Relationship between chronic exposure to ambient air pollution and mental health in Korean adult cancer survivors and the general population

**DOI:** 10.1186/s12885-021-09013-x

**Published:** 2021-12-04

**Authors:** Hyun-Jin Kim, Jin-young Min, Yong-Seok Seo, Kyoung-bok Min

**Affiliations:** 1 National Cancer Control Institute, National Cancer Center, Goyang, Republic of Korea; 2 Veterans Medical Research Institute, Veterans Health Service Medical Center, Seoul, Republic of Korea; 3 Young Jin Ind., Ltd., Icheon-si, Gyeonggi-do Republic of Korea; 4 Department of Preventive Medicine, College of Medicine, Seoul National University, 103 Daehak-ro, Jongno-gu, Seoul, 110-799 Republic of Korea

**Keywords:** Ambient air pollution, Chronic exposure, cancer, Depressive symptoms, Perceived stress, Suicidal ideation

## Abstract

**Background:**

Although a significant association between air pollution and mental health has been identified, few studies have addressed this relationship based on cancer diagnosis. This study investigated whether associations between long-term air pollution and mental health conditions differ based on whether the individual has been diagnosed with cancer.

**Methods:**

Nationally representative data were used and a total of 38,101 adults were included in the analyses. We assessed mental health factors such as perceived stress, depressive symptoms, and suicidal ideation, and analyzed the associations between these factors and individuals’ annual average exposure to air pollutants, including particulate matter with an aerodynamic diameter ≤ 10 μm (PM_10_), nitrogen dioxide, sulfur dioxide, and carbon monoxide.

**Results:**

Compared with the general population, PM_10_ exposure in cancer survivors predicted a higher risk of depressive symptoms (odds ratio [OR] =1.34; 95% confidence interval [CI] = 1.06–1.69) and suicidal ideation (OR = 1.29; 95% CI = 1.01–1.64). Notably, the statistically significant relationship between PM_10_ exposure and suicidal ideation in cancer survivors disappeared after further adjustment for depressive symptoms (*p* = 0.3103). This pattern was also observed in the result of propensity score-matched analysis for comparison between cancer survivors and the general population.

**Conclusions:**

This study provides the first evidence that cancer survivors with depressive symptoms may be more susceptible to suicidal ideation in the context of persistent PM_10_ exposure.

## Background

Psychological factors such as stress, depression, anxiety, and suicidal ideation represent major worldwide mental health burdens. Prolonged or repeated stress has been implicated in diseases such as obesity, cardiovascular disease, coronary heart disease, and certain cancers [1–4]. Moreover, depressive disorder increases suicide risk, which is a major cause of death [5] and most who die by suicide have comorbid mental health problems, such as extreme mood swings, anxiety, depression, and substance-related disorders [6]. Multiple factors, including the presence of pre-existing diseases and behavioral, biological, and various environmental components, may be intricately involved in the etiology of mental health problems.

The deleterious effects of air pollution on mental health are particularly noteworthy. Epidemiological studies have suggested a significant relationship between air pollution and mental health problems, such as perceived stress, depression, and suicide attempts [7–10]. Experimental studies in mice have provided insights into how air pollution exerts harmful effects on the central nervous system (CNS) [11–13]. These results highlight the possible pathway by which exposure to air pollution can trigger neurotoxic effects through oxidative stress, neuroinflammation, secondary inflammatory response via systemic circulation from the respiratory system, neurotransmitter alterations, and DNA damage [14].

Recently, interesting evidence has suggested that the association between suicide and air pollution is likely mediated by mental health conditions or comorbidities, including cardiovascular disease, stroke, and heart failure [15–17]. The observed synergistic effects of these comorbidities have not been fully elucidated; furthermore, patients with pre-existing diseases are more susceptible to air pollution exposure. In this study, we are concerned with cancer survivors. Cancer is one of the world’s largest health problems and ranks as the second leading cause of death following cardiovascular diseases [18]. Because cancer patients frequently experience mental illnesses, including psychological distress including psychological distress, anxiety, depression, and suicide before, during, or after treatment [19–22], they may be particularly vulnerable to air pollution-induced neurotoxic effects. However, to the best of our knowledge, no research has yet addressed whether the presence of cancer has an additional effect on the link between long-term air pollution exposure and poor mental health.

The aim of this study was to investigate the relationship between annual air pollution exposure and mental health (i.e., perceived stress, depressive symptoms, and suicidal ideation) in a nationwide sample of Korean adults and to identify whether these associations are mediated by a history of cancer diagnosis.

## Methods

### Study population

The study sample participated in the National Health and Nutrition Examination Survey (KNHANES) conducted by the Korean Centers for Disease Control and Prevention to assess the health and nutritional status of Koreans [23]. This nationwide cross-sectional survey was conducted using a multistage clustered probability sampling design and included information on demographic and socioeconomic status, health behaviors, anthropometric measures, clinical outcomes, and dietary intake. In total, 73,353 individuals participated in the fourth (2007–2009), fifth (2009–2012), and sixth (2013–2015) KNHANES waves. Our analyses included 38,101 participants who met the following inclusion criteria: (1) adults aged > 20 years; (2) those with residential region information that allowed the identification of air pollution exposure; (3) those with phenotypic psychological status information including perceived stress, depressive symptoms, and suicidal ideation; and (4) those who provided variables of interest, including target demographics and health-related behaviors. We classified the participants as cancer survivors if they had been diagnosed with cancer by a physician, regardless of cancer type. The study protocol was approved by the institutional review board of Seoul National University Hospital.

### Mental health outcomes: perceived stress, depressive symptoms, and suicidal ideation

Mental health information was obtained using a self-reported questionnaire. We evaluated three items as indicators of mental health: perceived stress, depressive symptoms, and suicidal ideation. Perceived stress status was assessed with the question, “How much stress do you feel in daily life?” to which the participants responded using a four-point Likert scale (1 = “very much,” 2 = “much,” 3 = “a little,” 4 = “almost none”). For the final analysis, we classified the participants into two groups: a stressed group who answered, “very much” or “much,” and an unstressed group who answered, “a little” or “almost none.” Depressive symptoms were identified using the question, “Have you been feeling depressed for over 2 weeks?” Possible answers were “yes” and “no.” Suicidal ideation was assessed using the question, “Have you ever thought about wanting to die in the last year?” Possible answers were “yes” and “no.”

### Measurement of air pollution exposure

Exposure to air pollution was assessed using atmospheric monitoring data collected at nationwide monitoring stations by the Ministry of the Environment of Korea (https://www.airkorea.or.kr). We obtained the annual average air pollutant concentration values, including particulate matter with an aerodynamic diameter ≤ 10 μm (PM_10_), nitrogen dioxide (NO_2_), sulfur dioxide (SO_2_), and carbon monoxide (CO) over a 9 year period (between January 1, 2007 and December 31, 2015) from each administrative division (i.e., seven metropolitan cities and nine provinces). Ambient air quality was measured in real time at approximately 280 fixed-site monitoring stations across the country. This study used a semi-ecological design that assigned the same level of exposure to all participants living in the same administrative district. Therefore, the annual averages of the fixed-site monitoring data measured in administrative district in which each participant resided were used to represent their exposure level to each ambient air pollutant. Of the 16 administrative divisions, one province (Jeju Island) was excluded from the analyses because of cultural and environmental differences.

### Other variables

To control for potential confounders, we investigated variables of interest including demographic characteristics and health-related behaviors. Demographic variables assessed by the questionnaire included age, sex, educational level, household income, and residence in an urban or rural location. Educational level was categorized into less than elementary school, middle school, high school, and college or graduate school. We used household income quartiles to control for the effect of income. Using the residential administrative districts, metropolitan regions and provinces were divided into urban and rural areas. Gyeonggi-do, which is adjacent to Seoul, the capital of South Korea, was classified as urban because it has recently undergone rapid urbanization (e.g., housing development and industrialization). Health-related behaviors such as smoking status, alcohol consumption, and moderate physical activity were also evaluated. Smoking status was categorized into three groups: never, former smoker, and current smoker. Alcohol consumption was categorized into four groups: never, less than once a month, two or three times a month, and more than four times a month [24]. We also obtained anthropometric data including height (m) and weight (kg), and we estimated body mass index (BMI) as kg/m^2^.

### Statistical analysis

We used Pearson’s correlation analysis to test the associations between the outcomes of interest and exposure levels to each of the four air pollutants. Multiple logistic regression analyses were conducted to identify associations between ambient air pollution and mental health in each subgroup (cancer survivors or the general population). The odds ratios (ORs) and 95% confidence intervals (CIs) for mental health outcomes were estimated, and converted to interquartile ranges (IQRs) according to the increase in the concentration of each air pollutant (9 μg/m^3^ for PM_10_, 11 ppb for NO_2_, 1 ppb for SO_2_, and 100 ppb for CO). Two adjustment models were evaluated to control for confounding variables. First, Model 1 was adjusted for demographic variables including age, sex, educational level, household income, and residence in an urban or rural location. Second, Model 2 was adjusted for these demographic variables plus health behaviors or conditions including smoking status, alcohol consumption, moderate physical activity, comorbidity (hypertension, diabetes, obesity, dyslipidemia, stroke, or myocardial infarction), and history of taking medications (antihypertensive drugs or diabetes drugs). We also assessed the relation to suicidal ideation after adjusting for depressive symptoms. In addition, to reduce the selection bias in each subgroup, association was compared using a propensity score matching (PSM) method. The propensity score was computed by a logistic regression including all the variables mentioned above as potential confounders. We performed 1:1 PSM with a caliper of 0.5 using the PSMATCH procedure in the SAS software (v. 9.4; SAS Institute, Cary, NC, USA). Subject’s characteristics were compared between cancer and general population before and after matching by using the t-test and chi-square test. All statistical analyses were conducted using SAS software (v. 9.4; SAS Institute, Cary, NC, USA) and *p* < 0.05 was considered statistically significant.

## Results

Table 1 shows the detailed characteristics of the cancer survivors and the general population before and after PSM. Before PSM, the cancer survivors had a slightly older mean age (61.2 years) (*p* < 0.0001) and a greater proportion of women (64.0%) (*p* < 0.0001) compared with the general population. The education level in cancer survivors was lower than that of the general population (*p* < 0.0001). About 67% of both cancer survivors and the general population resided in an urban area (*p* = 0.8639). The proportion of former smokers was higher among cancer survivors (28.5%) than in the general population (19.6%), but the proportion of current smokers was lower in the cancer survivors (8.6%) than among general population (21.6%). A total of 47.8 and 71.4% of cancer survivors and the general population reported drinking alcohol at least once a month, respectively. Except for obesity and stroke (both *p* > 0.05), cancer survivors had a higher proportion of comorbidity such as hypertension, diabetes, dyslipidemia, and myocardial infarction compared to those of general population (all *p* < 0.05). Cancer survivors who felt stressed and depressed in their daily lives represented 26.4 and 20.1%, respectively. About 20% of cancer survivors reported having thought about wanting to die in the past year. The distributions of basic characteristics after PSM were similar between cancer survivors and the general population (all *p* > 0.05).

**Table 1:** Baseline characteristics by cancer before and after propensity score (PS) matching

Characteristics	Before PS match			Before PS match		
	Cancer survivors	General population	*p*-value	Cancer survivors	General population	*p*-value
	Mean ± SD or n (%)	Mean ± SD or n (%)		Mean ± SD or n (%)	Mean ± SD or n (%)	
n	1279	36,822		1060	1060	
Age (years)	61.2 ± 12.7	49.6 ± 16.3	< 0.0001	60.3 ± 12.4	60.2 ± 13.7	0.7586
Female	818 (64.0)	21,169 (57.5)	< 0.0001	685 (64.6)	712 (67.2)	0.2161
Education level			< 0.0001			0.9963
≤ Elementary school	552 (43.2)	9715 (26.4)		439 (41.4)	440 (41.5)	
Middle school	193 (15.1)	3997 (10.9)		159 (15.0)	162 (15.3)	
High school	327 (25.6)	12,405 (33.7)		277 (26.1)	273 (25.8)	
≥ College/graduate school	207 (16.2)	10,705 (29.1)		185 (17.5)	185 (17.5)	
Residential region			0.8639			0.5786
Urban	857 (67.0)	24,757 (67.2)		707 (66.7)	719 (67.8)	
Rural	422 (33.0)	12,065 (32.8)		353 (33.3)	341 (32.2)	
Smoking			< 0.0001			0.5346
Current-smokers	110 (8.6)	7954 (21.6)		89 (8.4)	88 (8.3)	
Former-smokers	365 (28.5)	7215 (19.6)		291 (27.5)	269 (25.4)	
Never	804 (62.9)	21,653 (58.8)		680 (64.2)	703 (66.3)	
Alcohol Consumption(per month)			< 0.0001			0.8469
Never	667 (52.2)	10,520 (28.6)		525 (49.5)	543 (51.2)	
≤ 1	320 (25.0)	10,512 (28.6)		279 (26.3)	264 (24.9)	
2–3	234 (18.3)	13,139 (35.7)		206 (19.4)	206 (19.4)	
≥ 4	58 (4.5)	2651 (7.2)		50 (4.7)	47 (4.4)	
Moderate physical activity			< 0.0001			0.8897
Yes	407 (31.8)	14,260 (38.7)		350 (33.0)	347 (32.7)	
No	872 (68.2)	22,562 (61.3)		710 (67.0)	713 (67.3)	
Body mass index (kg/m^2^)	23.4 ± 3.3	23.7 ± 3.4	0.0087	23.5 ± 3.3	23.5 ± 3.0	0.9754
Comorbidity, n (%)						
Hypertension	518 (40.7)	11,315 (30.9)	< 0.0001	422 (39.8)	404 (38.1)	0.4228
Diabetes	188 (17.7)	3419 (10.0)	< 0.0001	187 (17.6)	169 (15.9)	0.2956
Dyslipidemia	135 (10.6)	2432 (6.6)	< 0.0001	116 (10.9)	119 (11.2)	0.8356
Obesity	381 (29.8)	11,703 (32.0)	0.1068	315 (29.7)	311 (29.3)	0.8490
Stroke	23 (1.8)	529 (1.4)	0.2874	17 (1.6)	17 (1.6)	1.0000
Myocardial infarction	53 (4.1)	773 (2.1)	< 0.0001	45 (4.3)	32 (3.0)	0.1313
Perceived stress						
Yes	337 (26.4)	9867 (26.8)	0.7222	271 (25.6)	230 (21.7)	0.0361
No	942 (73.7)	26,955 (73.2)		789 (74.4)	830 (78.3)	
Depressive symptoms						
Yes	257 (20.1)	5159 (14.0)	< 0.0001	208 (19.6)	154 (14.5)	0.0018
No	1022 (79.9)	31,663 (86.0)		852 (80.4)	906 (85.5)	
Suicidal ideation			< 0.0001			
Yes	249 (19.5)	5436 (14.8)		199 (18.8)	186 (17.6)	0.4639
No	1030 (80.5)	31,386 (85.2)		861 (81.2)	874 (82.5)	

The means and medians for the annual concentrations of each air pollutant are shown in Table 2. The average annual means (medians) of the PM_10_, NO_2_, SO_2_, and CO concentrations were 50.7 (49) μg/m^3^, 25.1 (24) ppb, 5.4 (5) ppb, and 549.0 (600) ppb, respectively. The IQRs for PM_10_, NO_2_, SO_2_, and CO concentrations were 9 μg/m^3^, 11 ppb, 1 ppb, and 100 ppb, respectively. There were significantly positive correlations between all air pollutants (all *p* < 0.05), with correlation coefficients ranging from 0.20–0.62.

**Table 2:** Air pollutants (annual average concentrations) and their distributions

Air pollutants	IQR	Mean (Median)	Pearson’s correlation coefficients			
			PM_10_	NO_2_	SO_2_	CO
PM_10_ (μg/m^3^)	9	50.7 (49)	1	0.43^***^	0.35^***^	0.62^***^
NO_2_ (ppb)	11	25.1 (24)	–	1	0.20^***^	0.34^***^
SO_2_ (ppb)	1	5.4 (5)	–	–	1	0.23^***^
CO (ppb)	100	549.0 (600)	–	–	–	1

*IQR* Interquartile range, *PM*_*10*_ Particulate matter < 10 μm in diameter, *NO*_*2*_ Nitrogen dioxide, *SO*_*2*_ Sulfur dioxide, *CO* Carbon monoxide

^*^p < 0.05, ^**^p < 0.01, ^***^p < 0.001

Table 3 shows the associations between air pollution and psychological factors stratified into cancer survivors and the general population, respectively. Among cancer survivors, no air pollution exposure measure was significantly associated with perceived stress (all *p* > 0.05), whereas all air pollutants except for SO_2_ were significantly associated with perceived stress in the general population (*p* < 0.05). Regarding depressive symptoms, only PM_10_ exposure in cancer survivors was significantly associated (OR, 1.34; 95% CI: 1.06–1.69), and the magnitude of their risk was greater than among the general population (OR, 1.11; 95% CI: 1.06–1.17). The analyses for suicidal ideation showed a similar pattern to that for depressive symptoms; i.e., the association between PM_10_ and suicidal ideation in cancer survivors (OR, 1.29; 95% CI: 1.01–1.64) was much stronger compared to that for the general population (OR, 1.15; 95% CI: 1.10–1.20). However, for PM_10_, the significant association with suicidal ideation disappeared after adjusting for depressive symptoms in Model 2 (*p* = 0.3103).

**Table 3:** Estimated associations of IQR increases in annual average air pollution and psychological factors in cancer survivors and general population before propensity score (PS) matching

	Cancer survivors (*n* = 1279)				General population (*n* = 36,822)			
	Model 1		Model 2		Model 1		Model 2	
	OR (95% *CI*)	*p*-value	OR (95% *CI*)	*p*-value	OR (95% *CI*)	*p*-value	OR (95% *CI*)	*p*-value
**Perceived stress**								
PM_10_ (μg/m^3^)	0.92 (0.76-1.11)	0.3653	0.98 (0.79-1.21)	0.8264	1.05 (1.02-1.10)	0.0016	1.06 (1.02-1.10)	0.0026
NO_2_ (ppb)	1.15 (0.84-1.59)	0.3878	1.32 (0.92-1.91)	0.1312	1.09 (1.03-1.16)	0.0029	1.10 (1.03-1.17)	0.0026
SO_2_ (ppb)	0.95 (0.84-1.07)	0.3935	0.96 (0.84-1.10)	0.5370	0.98 (0.96-1.01)	0.1322	0.99 (0.97-1.01)	0.3073
CO (ppb)	1.12 (0.96-1.31)	0.1590	1.19 (0.99-1.42)	0.0658	1.03 (1.01-1.06)	0.0164	1.03 (1.00-1.06)	0.0443
**Depressive symptom**								
PM_10_ (μg/m^3^)	1.28 (1.04-1.57)	0.0218	1.34 (1.06-1.69)	0.0139	1.11 (1.06-1.16)	< 0.0001	1.11 (1.06-1.17)	< 0.0001
NO_2_ (ppb)	1.10 (0.77-1.56)	0.6070	1.39 (0.93-2.07)	0.1100	1.12 (1.04-1.21)	0.0026	1.13 (1.04-1.23)	0.0028
SO_2_ (ppb)	0.94 (0.82-1.07)	0.3437	0.95 (0.82-1.10)	0.4720	1.01 (0.98-1.04)	0.4537	1.01 (0.98-1.04)	0.3814
CO (ppb)	1.15 (0.97-1.36)	0.1040	1.21 (1.00-1.48)	0.0563	1.05 (1.01-1.08)	0.0103	1.04 (1.01-1.08)	0.0267
**Suicidal ideation**								
PM_10_ (μg/m^3^)	1.36 (1.10-1.69)	0.0047	1.29 (1.01-1.64)	0.0459	1.15 (1.10-1.20)	< 0.0001	1.15 (1.10-1.20)	< 0.0001
NO_2_ (ppb)	1.25 (0.87-1.82)	0.2318	1.32 (0.87-2.00)	0.1912	1.07 (0.99-1.16)	0.0724	1.08 (0.99-1.17)	0.0698
SO_2_ (ppb)	0.97 (0.85-1.11)	0.6425	0.94 (0.81-1.10)	0.4497	1.02 (0.99-1.05)	0.1265	1.03 (1.00-1.06)	0.1065
CO (ppb)	1.20 (1.01-1.43)	0.0393	1.09 (0.89-1.33)	0.4274	1.03 (0.99-1.07)	0.0672	1.03 (0.99-1.06)	0.1617
**Suicidal ideation (adjustment for depressive symptom)**								
PM_10_ (μg/m^3^)	1.27 (1.00-1.62)	0.0492	1.16 (0.87-1.53)	0.3103	1.12 (1.07-1.18)	< 0.0001	1.12 (1.07-1.18)	< 0.0001
NO_2_ (ppb)	1.27 (0.84-1.91)	0.2621	1.18 (0.74-1.89)	0.4822	1.03 (0.95-1.12)	0.5263	1.03 (0.95-1.13)	0.4650
SO_2_ (ppb)	1.01 (0.87-1.17)	0.9336	0.97 (0.82-1.16)	0.7663	1.02 (0.99-1.05)	0.1743	1.02 (0.99-1.06)	0.1587
CO (ppb)	1.15 (0.95-1.39)	0.1679	0.98 (0.77-1.23)	0.8365	1.02 (0.98-1.05)	0.3937	1.01 (0.97-1.05)	0.5315

The odds ratio and 95% confidence interval in each air pollutant was scaled to the interquartile range for each pollutant, respectively (9 μg/m^3^ for PM_10_, 11 ppb for NO_2_, 1 ppb for SO_2_, and 100 ppb for CO)

Model 1 was adjusted for demographic variables including age, sex, education level, household income, and resident region (urbanity)

Model 2 was adjusted for demographic variables plus health behaviors including smoking status, alcohol drinking, moderate physical activity, comorbidity (hypertension, diabetes, obesity, dyslipidemia, stroke, or myocardial infarction), and history of taking medications (antihypertensive drugs or diabetes drugs)

*OR* Odds ratio, *CI* Confidence interval, *PM*_*10*_ Particulate matter < 10 μm in diameter, *NO*_*2*_ Nitrogen dioxide, *SO*_*2*_ Sulfur dioxide, *CO* Carbon monoxide

The associations between air pollution and psychological factors in cancer survivors and general population after PSM were demonstrated in Table 4. In cancer survivors, no air pollutant was significantly associated with perceived stress (all *p* > 0.05), whereas CO exposure in the general population had a significant association with perceived stress (OR, 1.25; 95% CI: 1.05–1.49). For depressive symptoms, a significant association with PM_10_ exposure was found only in cancer survivors (OR, 1.35; 95% CI: 1.08–1.68), not in the general population (OR, 0.98; 95% CI: 0.76–1.26). The results of suicidal ideation were similar to those of depression. Therefore, propensity score-matched analysis for comparison between cancer survivors and the general population showed that the association of PM_10_ exposure with depression or suicidal ideation is more pronounced in cancer survivors than in the general population.

**Table 4:** Estimated associations of IQR increases in annual average air pollution and psychological factors in cancer survivors and general population after propensity score (PS) matching

	Cancer survivors (*n* = 1060)		General population (*n* = 1060)	
	OR (95% *CI*)	*p*-value	OR (95% *CI*)	*p*-value
**Perceived stress**				
PM_10_ (μg/m^3^)	1.01 (0.83-1.24)	0.9120	1.03 (0.83-1.28)	0.8021
NO_2_ (ppb)	1.12 (0.88-1.41)	0.3556	1.16 (0.91-1.47)	0.2402
SO_2_ (ppb)	0.98 (0.86-1.12)	0.7751	1.14 (0.99-1.31)	0.0632
CO (ppb)	1.18 (0.99-1.40)	0.0624	1.25 (1.05-1.49)	0.0146
**Depressive symptom**				
PM_10_ (μg/m^3^)	1.35 (1.08-1.68)	0.0082	0.98 (0.76-1.26)	0.8504
NO_2_ (ppb)	1.16 (0.90-1.49)	0.2656	0.87 (0.65-1.15)	0.3231
SO_2_ (ppb)	0.97 (0.85-1.12)	0.7168	1.03 (0.87-1.21)	0.7429
CO (ppb)	1.21 (1.00-1.46)	0.0515	1.23 (1.00-1.52)	0.0475
**Suicidal ideation**				
PM_10_ (μg/m^3^)	1.30 (1.04-1.63)	0.0219	1.12 (0.89-1.41)	0.3459
NO_2_ (ppb)	1.10 (0.85-1.43)	0.4605	0.81 (0.62-1.06)	0.1226
SO_2_ (ppb)	0.97 (0.84-1.12)	0.7070	1.06 (0.91-1.24)	0.4206
CO (ppb)	1.06 (0.87-1.28)	0.5593	1.19 (0.98-1.44)	0.0781
**Suicidal ideation (adjustment for depressive symptom)**				
PM_10_ (μg/m^3^)	1.17 (0.91-1.51)	0.2305	1.16 (0.90-1.50)	0.2611
NO_2_ (ppb)	1.04 (0.77-1.39)	0.8078	0.83 (0.63-1.11)	0.2116
SO_2_ (ppb)	0.98 (0.83-1.15)	0.8137	1.07 (0.90-1.26)	0.4613
CO (ppb)	0.96 (0.77-1.19)	0.7101	1.12 (0.91-1.37)	0.2973

The odds ratio and 95% confidence interval in each air pollutant was scaled to the interquartile range for each pollutant, respectively (9 μg/m^3^ for PM_10_, 11 ppb for NO_2_, 1 ppb for SO_2_, and 100 ppb for CO)

*OR* Odds ratio, *CI* Confidence interval, *PM*_*10*_ Particulate matter < 10 μm in diameter, *NO*_*2*_ Nitrogen dioxide, *SO*_*2*_ Sulfur dioxide, *CO* Carbon monoxide

**Fig. 1:**
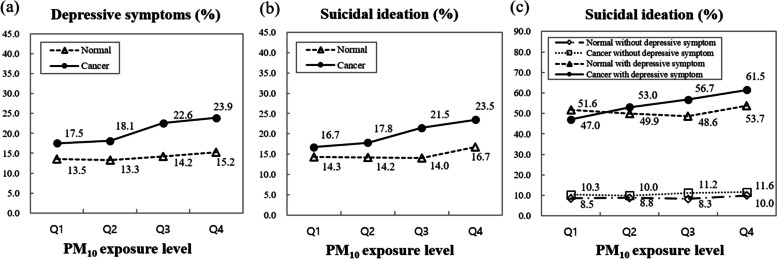
Percentage of subjects with depressive symptoms (**a**) or suicidal ideation (**b** and **c**) between cancer survivors and normal samples according to ambient PM_10_ exposure level (quartile1-quartile4)

As shown in Fig. 1, compared with the general population, cancer survivors showed a distinctly increased proportion with depressive symptoms or suicidal ideation with increasing quartiles of PM_10_ exposure. Specifically, as PM_10_ exposure levels increased, the rate of suicidal ideation in cancer survivors with depressive symptoms gradually increased compared with the general population.

## Discussion

This study comprehensively assessed the relationship between the annual average concentrations of ambient air pollutants and mental health outcomes, including perceived stress, depressive symptoms, and suicidal ideation, in Korean adults who were either cancer survivors or part of the general population. The association between air pollutants and perceived stress was significant in the general population, but not in cancer survivors. However, for depressive symptom and suicidal ideation, PM_10_ exposure in cancer survivors showed a relatively stronger association compared to the general population. Notably, the statistically significant relationship between PM_10_ exposure and suicidal ideation in cancer survivors disappeared after further adjustment for depressive symptoms. Therefore, the increased risk of suicidal ideation with PM_10_ exposure in cancer survivors may be closely related to depressive symptoms, given that the significance of the association between PM_10_ and suicidal ideation disappeared after adjusting for depressive symptoms. This pattern was also identified in the result of propensity score-matched analysis for comparison between cancer survivors and the general population.

Previous epidemiological studies have assessed the relations between air pollution and mental health. In 2015, a longitudinal study of older men identified a significant association between air pollution and a higher Perceived Stress Scale (PSS) score. In that study, there was a 3.2-point (95% CI: 2.1–4.3) increase in the PSS score per IQR increase in the 1-week moving average of particle number count [9]. Exposure to air pollution is also associated with maternal stress during pregnancy [25]. Lim et al. (2012) assessed the effect of air pollution on depressive symptoms in older adults and found that short-term exposure to PM_10_, NO_2_, and ozone was associated with depressive symptom changes, especially emotional symptoms [7]. A recent study also identified a significant association between fine particulate matter and depression and anxiety, which was strengthened by low socioeconomic status or the presence of comorbidities [17]. Other epidemiological studies of emergency department visits for depression have reported short-term effects of ambient air pollutant exposure on depression [26–28]. Most previous epidemiological studies on perceived stress and depression have focused on short-term exposure to air pollution, especially in older adults. Moreover, several studies in South Korea have identified deleterious effects of both short- and long-term exposure to air pollutants on suicide [15, 16, 29, 30]. However, recent results from Mexico City showed no evidence of an association between short-term air pollution exposure and daily suicides [31]. Similarly, a time series analysis showed no statistically significant associations between air pollution and suicide in four Colombian cities [32]. These inconsistencies in previous research on air pollution and suicide suggest that their relations may be complicated. Moreover, suicide research has predominantly focused on suicide attempts and deaths by suicide; thus, the need for further studies on suicidal ideation, a crucial precursor to later attempted or completed suicide, has been increasingly emphasized [8]. In this sense, our results may provide valuable evidence that the increased risk of suicidal ideation from PM_10_ exposure can lead to suicide attempts and completion.

Other recent evidence has emphasized the importance of comorbidities in the association between air pollution and suicide [15, 16, 31]. In 2010, Kim et al. showed that suicide risk from increased PM_10_ exposure was particularly intensified among participants with preexisting cardiovascular disease, although no significant association was found between short-term PM_10_ exposure and suicide in cancer patients [15]. A study of older adults in the United States also reported a significant association between fine particulate matter concentrations and depression or anxiety; this association was strengthened by low socioeconomic status and comorbidities including stroke and heart failure [17]. In addition, a large-scale national cohort study in South Korea reported that the risk for completed suicide due to long-term exposure to air pollution is higher among those with a physical disease or mental disorder [16]. Astudillo-García et al. also emphasized the mediating effect of comorbidities on the relations between air pollution and suicide [31]. Consistent with these previous studies, our results also demonstrate that participants who had received a cancer diagnosis were at a greater risk for suicidal ideation related to air pollution, particularly with PM_10_, exposure. Importantly, this pattern was more pronounced in cancer survivors with depressive symptoms compared to other subgroups. Cumulatively, our research supports the prevailing hypothesis that the link between air pollution and suicide is regulated by preexisting diseases or disorders.

Though the biological mechanisms linking air pollution and poor mental health remain unknown, previous studies have suggested several plausible explanations. First, air pollution may directly or indirectly affect inflammation pathways in the brain. Inflammation is a crucial risk factor in the etiology of CNS-related diseases [33]. Exposure to air pollution causes systemic inflammation in various organs, including the lungs and liver, which may affect CNS pathology through circulating proinflammatory cytokines such as TNF-α and IL-1β. In addition, inhaled particulate matter directly stimulates the proinflammatory response in the mouse brain [34], though no evidence of this has been reported for humans. Second, one experimental study of mice showed that exposure to particulates activates the hypothalamic–pituitary–adrenal (HPA) axis to release glucocorticoids [35]. Such HPA axis stimulation is involved in depressive disorders and stress response pathways [36, 37]. Finally, oxidative stress is recognized as a molecular mechanism involved in the pathogenesis of psychological disorders [38]. The overproduction of reactive oxygen species induced by ambient air pollution may cause oxidative stress, which may lead to mental health problems such as stress, depressive disorder, and suicide. However, more research is necessary to identify the full spectrum of mechanisms that may link ambient air pollution and mental conditions for patients with underlying comorbidities, especially cancer.

To our knowledge, this is the first large-scale study using national data to demonstrate an association between chronic exposure to air pollutants and mental health conditions based on the presence of a cancer diagnosis. However, this study is not without some limitations. First, we used a cross-sectional study design, which does not allow the determination of causal relations between air pollution and psychological status. Thus, these data cannot be used to determine whether individuals’ ambient air pollution exposure occurred before or after the onset of their mental health symptoms. Second, we used single items to assess self-reports of general psychological status, rather than clinical diagnoses or standardized questionnaires validated to quantify stress and depression levels using multiple scales. This is a disadvantage because we could not measure specific psychological symptoms using various scales. Recall bias may also have been present because our study required participants to remember over a long period of time instead of recording a clinical diagnosis. Third, when estimating individuals’ exposure to air pollution, we could not assess factors such as the proximity of their house to the road or workplace exposure since this information was not included in the original survey. In addition, an individual’s exposure to air pollutants can vary between their home and other places they visit, and therefore this could introduce exposure measurement error. Lastly, we could not determine an association with PM_2.5_, which may have more detrimental health effects than PM_10_ [39, 40], due to the lack of relevant data.

## Conclusions

Our study used a large-scale national dataset of Korean adults to show that cancer survivors are more susceptible to depressive symptoms and suicidal ideation in association with persistent PM_10_ exposure. Furthermore, these strong associations observed in cancer survivors may be explained by depressive symptoms. Additional research on specific cancer types and clinical stages is needed to better explain the associations between air pollution exposure, mental health, and cancer.

## Data Availability

The datasets analyzed during the current study are available in website of The Korea Disease Control and Prevention Agency; Korea National Health and Nutrition Examination Survey repository, [https://knhanes.kdca.go.kr/knhanes/sub03/sub03_02_05.do].

## Abbreviations

CNS: Central nervous system
KNHANES: The National Health and Nutrition Examination Survey
PM_10_: Particulate matter with an aerodynamic diameter ≤ 10 μm
NO_2_: Nitrogen dioxide
SO_2_: Sulfur dioxide
CO: Carbon monoxide
BMI: Body mass index
ORs: Odds ratio
Cis: Confidence intervals
IQRs: Interquartile ranges
PSS: Perceived Stress Scale
HPA: Hypothalamic–pituitary–adrenal
